# Pseudocyst in Neck: A Case Report on Rare Complication of Ventriculoperitoneal Shunt

**DOI:** 10.1155/2021/6656506

**Published:** 2021-04-30

**Authors:** B. Nitin, Manish Gupta, Anshul Singh

**Affiliations:** Department of ENT, Maharishi Markandeshwar Institute of Medical Sciences & Research,MMDU, Ambala, India

## Abstract

**Introduction:**

Ventriculoperitoneal shunt is a common neurosurgical procedure, for the definitive management of hydrocephalus. Shunt failures may occur due to various causes but are usually due to infections in adults and catheter occlusion in the paediatric population. *Case Report*. The 13-year-old girl presented with a right lateral neck swelling. In detailed history, she was found to be an old case of ventriculoperitoneal shunt. The clinical examination and radiological investigation revealed proximal dislodgment of the stent from the cranium, causing persistent cerebrospinal fluid (CSF) leak and pseudocyst formation in the neck.

**Conclusion:**

The case highlights CSF pseudocyst formation as a rare differential for lateral neck swellings.

## 1. Introduction

Ventriculoperitoneal (VP) shunt is surgically placed in patients with hydrocephalus [1]. Due to the increased life expectancy of such patients, there are a variety of delayed complications [2]. Postoperative delayed obstruction of the shunt and intracranial infection is the leading complication, while the pseudocyst formation, allergy to shunt material, shunt migration, segmental disconnection, breakage, bowel perforation, and overdraining of cerebrospinal fluid (CSF) leading to subdural hematoma are rarely seen [3, 4].

Pseudocysts get formed due to the collection of CSF fluid, usually accumulating around the distal end of the shunt and have a wall of fibrous tissue, thus devoid of the epithelium as seen in cyst [5]. In CSF pseudocyst, the CSF collects in the subcutaneous plane and often causes surrounding inflammation, due to shunt infection or following multiple surgeries [6, 7]. In a detailed English literature search, only few cases of pseudocyst formation in the abdomen were found.

Here, we present a case of a very rare VP shunt complication, CSF pseudocyst in the neck. However, it is second in world English literature, but very first to report proximal end migration of VP shunt [1].

## 2. Case Report

The 13-year-old girl presented in our outpatient department with a complaint of right-sided neck swelling for four months. It was insidious in onset, painless, and was gradually progressive. There was no history of fever, restriction of head neck movement, or sudden change in size. Her parents gave a history of hydrocephalus at birth, for which she was operated on at 6 months of age. There was no history of headache, vertigo, or any neurological deficit. History of any head-neck trauma prior to the swelling was negative. The previous surgery details were not available, and thus, the knowledge of shunt material used or whether it was tied to the pericranium was not known. There was no history of any shunt revision surgery after the first operation.

**Figure 1:**
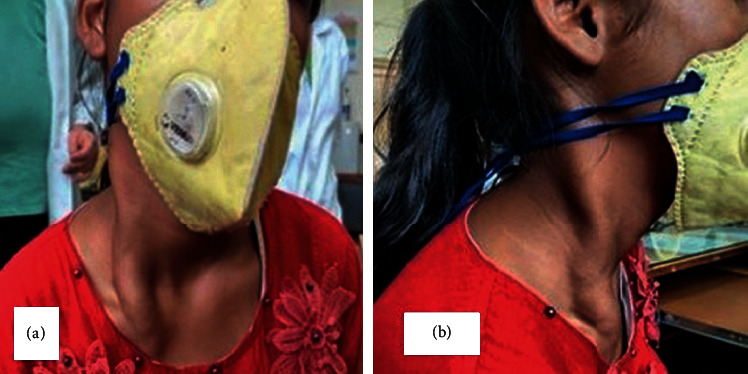
(a, b) Anterior and lateral clinical picture, showing tortuous swelling overlying right sternocleidomastoid.

On examination of the patient, the swelling of 12 cm × 4 cm was observed on the right lateral neck (Figures 1(a) and 1(b)). The swelling extended from the right mastoid tip, along the sternocleidomastoid muscle to one finger below the clavicle on the anterior chest wall. The swelling was tortuous with no dilated veins present over it. The skin over the swelling was normal, with no signs of inflammation or puckering seen.

**Figure 2:**
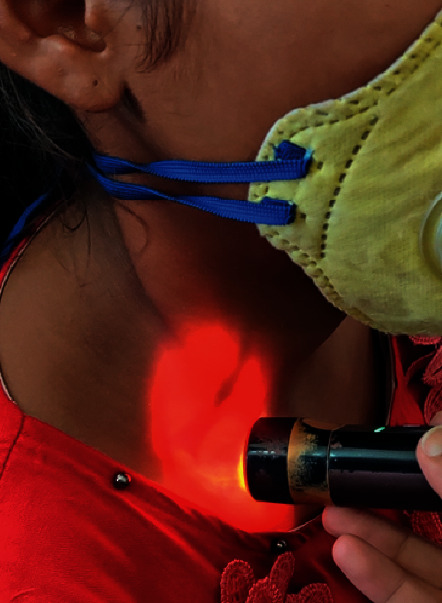
Positive transillumination test of the patient with the shadow of shunt in situ.

On palpation, it had a normal temperature and was nontender. It was well defined, cystic, compressible but not reducible or fluctuant. A cord-like structure was felt running from the postauricular region to the swelling. The cord was hard in consistency not fixed to underlying tissue, as was mobile side to side. No pulsations were felt in and around the swelling. The transillumination test was positive with a brilliant glow of the swelling (Figure 2) with the shadow of a cord-like structure. No bruit was heard on auscultation.

The ultrasonography (USG) of the neck revealed the intracervical part of the VP shunt with perishunt large fluid collection. Fine-needle aspiration was done in our outpatient department, under aseptic measures which revealed clear colourless thin aspirate. The fluid sent for biochemical analysis confirmed CSF with glucose: 73 mg/dL and proteins: 45 mg/dL. The fluid was also sent for culture and sensitivity, which gave no growth at 48 hours.

**Figure 3:**
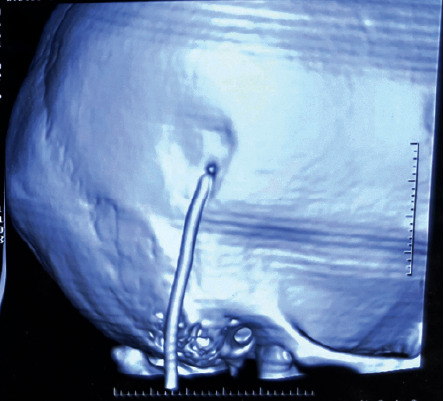
The 3-dimensional reconstructed image of CT scan showing VP shunt proximal end lying extracranially.

**Figure 4:**
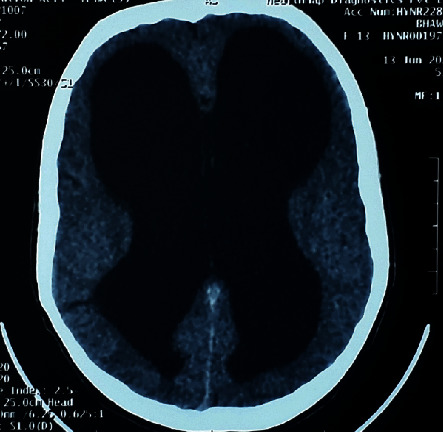
Computed tomography of the brain, axial section showing dilated ventricles suggestive of hydrocephalus.

**Figure 5:**
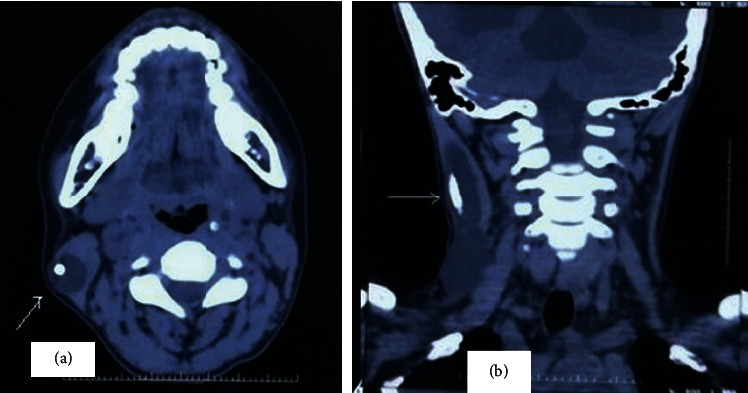
(a, b) Noncontrast computed tomogram of neck, coronal and axial sections showing the CSF pseudocyst involving the sternocleidomastoid on the right side with VP shunt in situ (white arrow).

The patient was advised for a computed tomography scan (CT) to look for the position of the shunt intracranially and possible changes in the brain due to increased intracranial pressure following shunt dysfunction. Because of the availability of CT, its high sensitivity in comparison with plain X-ray, and better delineation of neck mass, the plain X-ray (shunt series) was not asked for in our case. On the CT brain, there was no evidence of shunt intracranially, its proximal end lying just external to the cranium was present (Figure 3), and hydrocephalus was present (Figure 4). The CT neck revealed a cystic lesion, within the right sternocleidomastoid with a radiopaque shunt lying within (Figure 5).

The patient was then referred to the neurosurgery department for VP shunt revision surgery. After written informed consent was obtained from the parents, the shunt was replaced and successfully sutured to the pericranium on the same side in the posterior parietal part of the cranium and the peritoneal cavity. The pseudocyst resolved, with no recurrence in one-year follow-up.

## 3. Discussion

Hydrocephalus, as the name suggests, is a condition of the brain following excessive accumulation of CSF [8]. This leads to increased intracranial tension and manifests in adults with headache, double vision, body imbalance, urinary incontinence, and personality changes with poor mental function. In children and newborns, it may present with a rapid increase in the size of the head with vomiting, drowsiness, seizures, or exophthalmos. While a number of methods have been used to categorize hydrocephalus, Walter Dandy's classification of hydrocephalus as either communicating or noncommunicating remains central to surgical decision-making. There is no universally accepted classification system for hydrocephalus [9]. There are various aetiologies, which include haemorrhage, infection, tumour, and congenital anomalies, or they can be idiopathic [10].

In our patient, despite a nonfunctioning shunt, no symptoms were seen, due to continuous trickle of CSF through the cranium opening into the neck subcutaneous plane. Lateral neck swellings in children are mostly thought to be reactive lymphadenopathy, lymphadenitis (viral and bacterial), lymphangioma, branchial cleft cyst, and vascular malformation [11].

The use of VP shunt in hydrocephalus patients has almost replaced the ventriculoatrial (VA) shunt due to the fewer incidences of infection and clotting [12]. The various parts of the shunt are ventricular catheter, reservoir (valve), and long distal catheter. During placement, the distal catheter is tunnelled beneath the skin of the scalp, neck, and chest to reach the peritoneal cavity [13]. Due to the availability of better medical aid, the patients with VP shunt have a long life, which actually had an increased variety of delayed complications [2]. According to the literature, the reported incidence of complications following VP shunt surgery varies between 24 and 47%, of which the majority are abdominal (25%) [8]. The complications following shunt surgery have been classified as infective, mechanical or functional, and neurological or nonneurological [8, 14, 15]. Early shunt infections are caused by contamination due to normal skin flora during shunt placement, and late infections are due to haematogenous route, peritonitis, bowel perforation, or abdominal pseudocyst [13, 16]. Also, few complications associated with VP shunts breakage, obstruction, migration, or mispositioning have been reported. Rarely distal end may migrate and penetrate the stomach wall, cause gall bladder or urinary bladder injury, perforate into vagina or bowel, or present with umbilical CSF fistula or cervical myelopathy [14, 17, 18]. These complications can be encountered either in the immediate perioperative, postoperative, or on follow-up. Further, the VP shunt distal end may migrate in the thorax, causing fluid, air, or both collection and respiratory distress [19]. Clinically, patients may have headache and vomiting due to shunt dysfunction and fever following infection [20, 21]. The pseudocyst formation within the abdomen may present with a palpable mass, distension, and pain [22, 23]. Although the exact pathophysiological mechanism underlying the formation of abdominal pseudocysts is unknown, several predisposing factors for cyst formation have been reported in children and adults, including changes in the absorption of CSF secondary to inflammatory or infectious processes in the peritoneum, peritoneal adhesions from previous abdominal surgery, and increase in the protein content of CSF [7]. There is one case in the literature, reporting retrograde migration of VP shunt distal end into the neck [15]. This displacement is often due to the abrupt release of the anchoring point following fibrotic scar formation. Also, the continuous flow of CSF from the functioning catheter eases the upward movement of the shunt [1]. Similarly, in another case, being reported in the literature, the relatively shorter intra-abdominal length of the peritoneal catheter and unintentional subcutaneous tunnelling caused extrusion of the peritoneal end of the catheter [1]. Further, the skin in the supraclavicular region is loose as compared to thoracic skin; thus, any leak may collect in the neck subcutaneously forming CSF hygroma [1].

But, in our case, first one in literature, there was dislodgment of the ventricular end (proximal end) of the shunt, which led to extravasation of CSF extracranially. This persistent fistula in the cranial vault of the child resulted in CSF pseudocyst of the neck. As there was free flow of CSF in the neck, our patient had no complaints of headache, vertigo, or vomiting.

### 3.1. Main Points

  CSF pseudocyst formation in the neck after VP shunt surgery is very rare
  This case adds an important differential for lateral neck cystic swelling
  The proximal end of VP shunt may also get dislodged, and on regular follow-up of the patient after VP shunt surgery, this point in mind may avoid such complication

## 4. Conclusion

It is rare to see CSF pseudocyst in the neck after VP shunt surgery. The case presented here adds another differential for lateral neck swelling and further stresses the need for detailed history.

## Data Availability

Datasets were derived from public resources and made available with the article at locations cited in the reference section. We are aware the data underlying the findings of this paper will be publicly available and allow the same for use wherever possible.

## References

[1] Chopra S., Singh D. K., Kumar B., Gupta A., Gupta V. (2009). CSF hygroma in the neck: rare complication of ventriculoperitoneal shunt. *Pediatric Neurosurgery*.

[2] Diyora B., Bhende B., Dhall G., Kamble H., Nayak N. (2019). Subcutaneous cerebrospinal fluid pseudocyst: an unusual complication of ventriculoperitoneal shunt. *Journal of Neurosciences in Rural Practice*.

[3] Paff M., Alexandru-Abrams D., Muhonen M., Loudon W. (2018). Ventriculoperitoneal shunt complications: a review. *Interdisciplinary Neurosurgery*.

[4] Ezzat A. A. M., Soliman M. A. R., Hasanain A. A. (2018). Migration of the distal catheter of ventriculoperitoneal shunts in pediatric age group: case series. *World Neurosurgery*.

[5] Tamura A., Shida D., Tsutsumi K. (2013). Abdominal cerebrospinal fluid pseudocyst occurring 21 years after ventriculoperitoneal shunt placement: a case report. *BMC Surgery*.

[6] Anderson C. M., Sorrells D. L., Kerby J. D. (2003). Intraabdominal pseudocysts as a complication of ventriculoperitoneal shunts. *Journal of the American College of Surgeons*.

[7] Ohba S., Kinoshita Y., Tsutsui M. (2012). Formation of abdominal cerebrospinal fluid pseudocyst. *Neurologia Medico-Chirurgica*.

[8] Ghritlaharey R. K., Budhwani K. S., Shrivastava D. K. (2007). Trans-anal protrusion of ventriculo-peritoneal shunt catheter with silent bowel perforation: report of ten cases in children. *Pediatric Surgery International*.

[9] Rekate H. L. (2009). A contemporary definition and classification of hydrocephalus. *Seminars in Pediatric Neurology*.

[10] Cherian S., Whitelaw A., Thoresen M., Love S. (2004). The pathogenesis of neonatal post-hemorrhagic hydrocephalus. *Brain Pathology*.

[11] Meier J. D., Grimmer J. F. (2014). Evaluation and management of neck masses in children. *American Academy of Family Physicians*.

[12] Andreoli S. P. (2010). Glomerulonephritis in children. *Pediatric Urology*.

[13] Vinchon M., Lemaitre M. P., Vallée L., Dhellemmes P. (2002). Late shunt infection: incidence, pathogenesis, and therapeutic implications. *Neuropediatrics*.

[14] Borkar S. A., Satyarthee G. D., Khan R. N., Sharma B. S., Mahapatra A. K. (2008). Spontaneous extrusion of migrated ventriculoperitoneal shunt catheter through chest wall: a case report. *Turkish Neurosurgery*.

[15] Felipe-Murcia M., Almagro M. J., Martínez-Lage J. F. (2006). Retrograde migration of ventriculoperitoneal shunt to the neck. case report. *Neurocirugia*.

[16] Baird C., O’Connor D., Pittman T. (1999). Late shunt infections. *Pediatric Neurosurgery*.

[17] Mohindra S., Singla N., Gupta R., Gupta S. K. (2007). CSF fistula through the umbilicus following a shunt surgery: a case report and literature review. *Pediatric Neurosurgery*.

[18] Wolfe S. Q., Bhatia S., Green B., Ragheb J. (2007). Engorged epidural venous plexus and cervical myelopathy due to cerebrospinal fluid overdrainage: a rare complication of ventricular shunts—case report. *Journal of Neurosurgery*.

[19] Akyüz M., Uçar T., Göksu E. (2004). A thoracic complication of ventriculoperitoneal shunt: symptomatic hydrothorax from intrathoracic migration of a ventriculoperitoneal shunt catheter. *British Journal of Neurosurgery*.

[20] Drake J. M., Kulkarni A. V. (1993). Cerebrospinal fluid shunt infections. *Neurosurgery Quarterly*.

[21] Mcclinton D., Carraccio C., Englander R. (2001). Predictors of ventriculoperitoneal shunt pathology. *Pediatric Infectious Disease Journal*.

[22] Aparici-Robles F., Molina-Fabrega R. (2008). Abdominal cerebrospinal fluid pseudocyst: a complication of ventriculoperitoneal shunts in adults. *Journal of Medical Imaging and Radiation Oncology*.

[23] De Oliveira R. S., Barbosa A., Vicente Y. A. D. M. V. D. A., Machado H. R. (2007). An alternative approach for management of abdominal cerebrospinal fluid pseudocysts in children. *Child’s Nervous System*.

